# Surface Characterization, Antimicrobial Activity of Nonthermal Atmospheric-Pressure Plasma Jet on Polyvinyl Siloxane Impression Materials

**DOI:** 10.3390/medicina58111556

**Published:** 2022-10-29

**Authors:** Yu-Ri Choi, Eun-Mi Yoo, Hye-Yeon Seo, Min-Kyung Kang

**Affiliations:** 1 Department of Dental Hygiene, Hallym Polytechnic University, Chuncheon 24210, Korea; 2 Department of Dental Hygiene, Hanyang Women’s University, 200, Salgoji-gil, Seoul 04763, Korea; 3 Department and Research Institute of Dental Biomaterials and Bioengineering, Yonsei University College of Dentistry, 50 Yonsei-ro, Seoul 03722, Korea; 4 Department of Dental Hygiene, Hanseo University, Seosan 31962, Korea

**Keywords:** nonthermal atmospheric-pressure plasma jet, dental polyvinyl siloxane impression material, disinfection

## Abstract

*Background and Objectives* The antimicrobial efficacy of a nonthermal atmospheric-pressure plasma jet (NAPPJ) on dental impression materials was investigated. *Materials and Methods* Type 3 polyvinyl siloxane was used as the impression material, and air and nitrogen NAPPJ were applied. The antibacterial effect of the NAPPJ was measured using the number of colony-forming units (CFUs) and scanning electron microscopy (SEM) images of *Streptococcus mutans*. Surface chemical characteristics of the impression material were examined using X-ray photoelectron spectroscopy (XPS) and contact angle measurement. Additionally, physical properties were analyzed through surface roughness measurement, detail reproduction, and strain-in-compression test. *Results* Compared with the control group, the plasma treatment group showed ruptured bacteria membranes, destroyed bacteria structures, a significant reduction in the number of CFUs, and a significantly reduced contact angle. Further, XPS analysis showed that their surface was significantly richer in hydroxyl groups. The surface roughness, detail reproduction, and strain-in-compression results indicated no significant differences between the plasma treatment and control groups. NAPPJ treatment could remove bacteria from polyvinyl siloxane dental impression materials without changing the surface's physical properties. *Conclusion* Therefore, it is considered a promising method for disinfection.

## 1. Introduction

Dental rubber impression materials are used for accurately replicating the details of soft and hard tissues in the oral cavity. Polyvinyl siloxane has been widely used as a dental impression material owing to its excellent physical properties and handling properties [1].

Dental clinics are susceptible to the transfer of infectious oral biofilms from patients to dental workers or vice versa and even from patient to patient through saliva, blood, and secretions [2]. Cross-infections are a major issue in dentistry. In particular, caution is required during the impression-taking process, because impressions are always covered with saliva or blood that may contain potentially pathogenic microorganisms or the hepatitis B or C or herpes simplex viruses. To eliminate cross-infections, impression materials must be washed in running water and treated with disinfectants [1,3,4,5]. Commonly, Dental elastomeric impression materials are immersed in or sprayed with disinfectants such as sodium hypochlorite, glutaraldehyde, or povidone–iodine in the dental clinic. However, disinfectants stimulate the skin. Therefore, dental workers need to be careful when handling disinfectants during the disinfection procedure. In addition, some disinfectants change the physical properties of impression materials [2,6].

Therefore, there is a need for an alternative disinfection method for impression materials. Recently, atmospheric-pressure plasmas have been used in medical applications. The nonthermal atmospheric-pressure plasma jet (NAPPJ) exhibits a lower degree of ionization and the reaction mixture is far from achieving thermal equilibrium. It can be used effectively at low temperatures and does not have any special quenching requirements. Hence, non-thermal plasma is ideal for biomedical applications [7]. The antibacterial characteristics of NAPPJ have been studied for their application in implants, dental instruments, and toothbrushes [8,9,10]. Further, NAPPJ is known to be harmless to the human body, provide bactericidal effects, and have the possibility of being applied to exposed areas without damaging cells [9,11]. Previous studies commonly used air, nitrogen, helium, and other gases for antibacterial effects. Especially, compressed air was desirable because that was inexpensive and easily available in the dental clinic [8,12]. However, in dentistry, their use has thus far been limited to applications such as root canal sterilization [13,14,15]. Furthermore, there have been few studies on the disinfection effect of impression materials using NAPPJ and their effect on the physical and chemical properties of impression materials. Therefore, it is unclear whether chemical changes induced by NAPPJ would cause inhibition of bacteria on the impression surface. 

In this light, the present study was to evaluate the antimicrobial effect on *S. mutans* to investigate the utility of NAPPJ for disinfecting polyvinyl siloxane impressions, which is a commonly used impression material for dental clinics and analyze its effects on the physical and chemical properties of the impressions. The null hypothesis was as follows: (1) there is no difference in the chemical change in the impression surface after NAPPJ, and (2) there is no difference in the antimicrobial effects against *S. mutans* after NAPPJ.

## 2. Materials and Methods

### 2.1. Sample Preparation

The polyvinyl siloxane impression material investigated (Imprint II; 3M ESPE) is a Type 3 material according to ISO 4823. To make the impression specimens, a Teflon mold with a diameter of 15 mm and thickness of 2 mm was placed on a glass plate. The polyvinyl siloxane material was placed inside the mold and pressed with another glass plate from the top to produce a disc-shaped specimen. The impression material was allowed to set according to the setting time of the manufacturer. Each group for testing each physical property (e.g., contact angle measurement, surface roughness, detail reproduction test, strain-in-compression tests) consisted of 10 specimens.

### 2.2. NAPPJ Treatment

**Figure 1:**
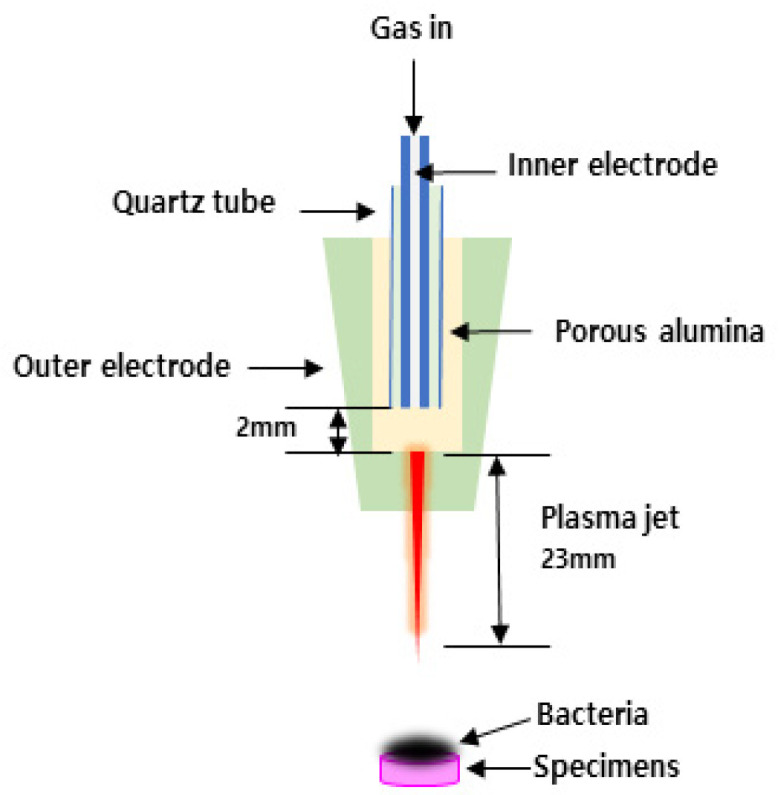
Schematic diagram of non-thermal atmospheric pressure plasma treatment on the impression materials.

The NAPPJ equipment was designed and provided by Kwangwoon University (Plasma Bioscience Research Center, Kwangwoon University, Seoul, Korea). Air and nitrogen were used as plasma gases, and the gas flow rate was 1 L/min. Each sample was placed on a pedestal. The distance between the tip of the NAPPJ frame and the sample was 5 mm. Each of the test samples was treated with the NAPPJ for 10 min. The control group was not treated with plasma (Group C). The experimental groups included a nitrogen-plasma-treated group (Group N) and an air-plasma-treated group (Group A). The voltage and current values used for operating the plasma were 15 kV and 13 mA, respectively. The details of the plasma source can be found elsewhere [16]. The antibacterial effect of NAPPJ was evaluated. Firstly, *S. mutans* were formed on the impression materials. Additionally, then NAPPJ was applied, respectively (Figure 1). 

### 2.3. Microbial Culture

Gram-positive *Streptococcus mutans* (*S. mutans* ATCC 25175) was acquired from the Korean Culture Center of Microorganisms (KCCM). *S. mutans* was cultured using brain heart infusion (BHI; Becton Dickinson and Company, USA) at 37 °C. 

The specimens were placed in the middle of a Petri dish (SPL, 35 mm × 1 mm), and 100 µL of the bacteria suspension (1 × 10^5^) was seeded on the specimens.

### 2.4. Evaluation of Bacterial Colony Forming Units (CFUs)

After each NAPPJs treatment, the specimens were incubated for 24 h at 37 °C. They were then transferred to a 24-well plate, and 1 mL of culture medium was inserted into each well.

The bacteria were suspended using a sonicator (Ultrasonic cleaner SH-2100, Saehan Ultrasonic Co., Seoul, Korea) for 1 min. Then, 100 µL of the suspension was seeded on a solid agar plate for 24 h, and the CFU of the bacteria was counted.

### 2.5. Morphology of S. mutans

To observe the morphological change in bacteria, the specimens were inoculated with bacteria (1 × 10^5^ /100 µL) and after each plasma treatment, the specimens were inserted with paraformaldehyde for fixation, and the specimens were rinsed with PBS. Then, it was observed with a Field-emission scanning electron microscope (Fe-SEM; S-3000N, Hitachi Co., Tokyo, Japan). Imaging with an acceleration voltage of 20 kV was performed at 500× magnification.

### 2.6. Surface Chemistry

The surface chemistry of the specimens was analyzed using X-ray photoelectron spectroscopy (XPS; K-alpha, Thermo VG, UK). Monochromatic Al Kα was used as the X-ray source (Al Kα line: 1486.6 eV). Plasma irradiation was measured in reference to the C1s peak at 284.8 eV. The X-ray beam power was relatively calibrated, and measurements were performed at 12 kV, 3 mA, with a spot diameter of 400 μm. The carbon and oxygen contents were examined to evaluate the change in the surface chemistry.

### 2.7. Contact Angle Measurement

The wettability of the specimen was determined using a contact angle measurement system (Phoenix 300, SEO Co., Korea) by dropping 4 µL of distilled water at the center of each sample. The falling droplet was captured using a camera and image analysis program (Image XP, SEO Co., Suwon, Korea) after 5 s. Additionally, the static contact angle was measured. The average values on the right and left sides were determined as a result. 

### 2.8. Surface Roughness

The surface roughness of all specimens was measured before and after NAPPJ treatment. The surface roughness was evaluated using a noncontact optical surface roughness tester (GT-X3 BASE, Brucker Co., Bremen, Germany). The observation mode was set to VSI using a green luminous source, and an area of 63 mm × 47 mm was tested. 

### 2.9. Strain-in-Compression Tests

For strain-in-compression tests, cylindrical specimens of the control and plasma treatment groups with a diameter of 10 mm and a maximum height of 20 mm were prepared according to ISO 4823.

### 2.10. Detail Reproduction Test 

Three specimens were made according to ISO 4823. Polyethylene sheets were placed in a water bath maintained at (37 ± 1) °C for 4 min to simulate the temperature environment in the mouth; then, they were placed on a flat glass (5 m × 5 m) for 60 s. Next, the specimens were blow-dried, following which the image analyzer (Hirox KH-1000, Hirox Co., Tokyo, Japan) was used to examine the specimen.

### 2.11. Statistical Analysis

Statistical analysis software (SPSS, PASW Statistics 18.0, IBM Inc., Armonk, NY, USA) was used to evaluate significant differences in the physical properties for all groups with one-way analysis of variance (ANOVA). This software was applied for the surface roughness measurement, contact angle measurement, and strain-in-compression tests. Tukey’s test was performed (*p* = 0.05) to verify the one-way ANOVA results.

## 3. Results

### 3.1. CFUs of S.mutans

**Figure 2:**
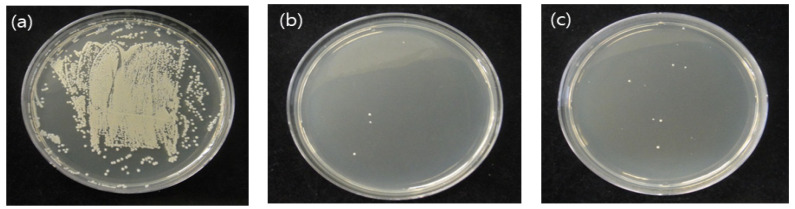
CFU of *S. mutans*: (**a**) control group, (**b**) nitrogen-plasma-treated group, and (**c**) air-plasma-treated group.

Figure 2 shows the antibacterial activity results. The CFUs of the control, nitrogen, and air groups were 720 ± 35, 4.3 ± 2.3, and 13.6 ± 8, respectively. The number of bacteria was significantly reduced in all experimental groups compared with that in the control group (*p* < 0.05). Further, no significant differences were seen between the experimental groups.

### 3.2. Morphology of S. mutans

**Figure 3:**
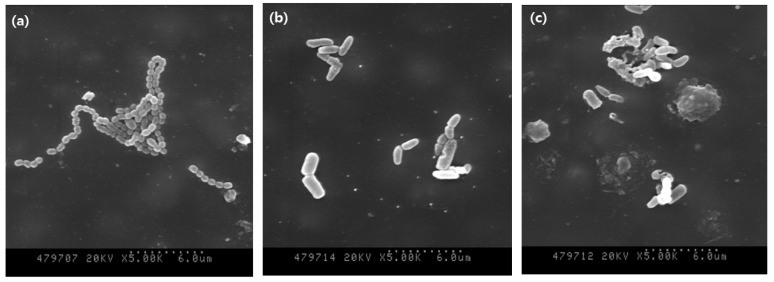
Field-emission scanning electron microscope images of *S. mutans*: (**a**) control group, (**b**) nitrogen-plasma-treated group, and (**c**) air-plasma-treated group.

Figure 3 shows the morphologies of the microorganisms. The number of microorganisms was confirmed to be decreased in the NAPPJ-treated groups compared with that in the non-treated control group. In addition, the morphology of *S. mutans* revealed that the chain of bacteria was destroyed, and the shape became irregular.

### 3.3. Surface Chemistry

**Figure 4:**
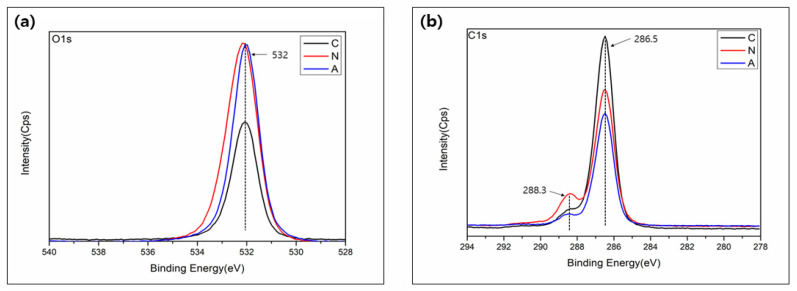
XPS measurement spectra on surface of specimens: (**a**) O1s and (**b**) C1s.

The chemistry change in the polyvinyl siloxane impression material was analyzed by XPS. As shown in Figure 4a, a hydroxyl group (OH^−^) at 532 eV was observed. Additionally, more OH^-^ groups were seen with both nitrogen and air plasma treated groups compared to no treated control group. Further, Figure 4b shows two peaks corresponding to the carbon-hydrogen-carbon group (C-O-C) at 286.5eV and the carboxyl group (COOH) at 288.3 eV. The C_1_ peak (286.5 eV) showed a small decrease after plasma treatment.

### 3.4. Contact Angle Measurement

**Figure 5:**
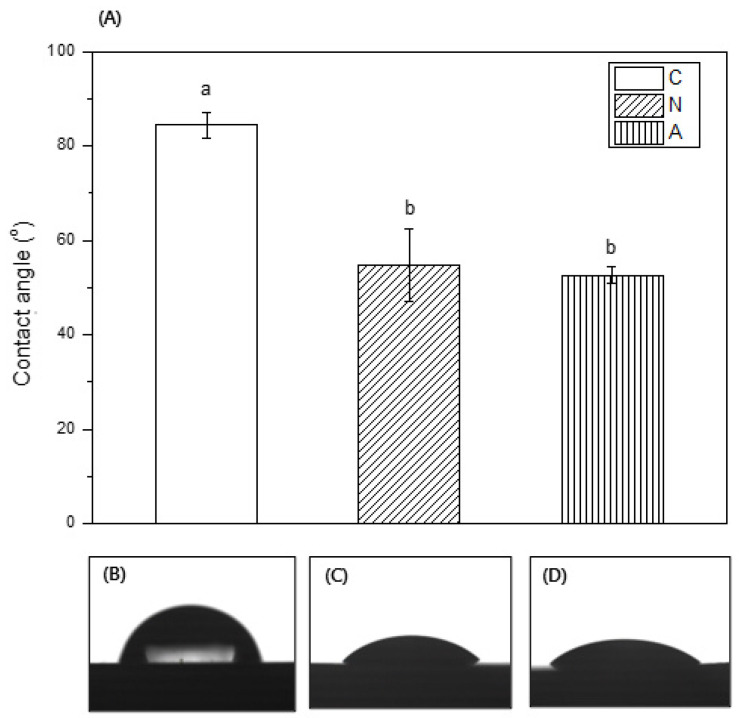
Wettability measurement result of the specimen: (**A**) contact angle measurement result graph. C: control (non-treated) group, N: nitrogen-plasma-treated group, and A: air-plasma-treated group. Significant differences were seen between the control and the experimental groups (*p* < 0.05). (**B**–**D**) Water droplet mages (**B**) control group, (**C**) nitrogen-plasma-treated group, (**D**) air-plasma-treated group.

The wettability of the specimen was evaluated through a contact angle observation; Figure 5 shows the contact angle results. The contact angles of the control, nitrogen, and air groups were 84.41° ± 2.75°, 54.84° ± 7.75°, and 52.58° ± 1.75°, respectively. All plasma-treated groups showed a significantly smaller contact angle, compared with that of the control group (*p* < 0.05). Thus, the plasma-treated experimental groups became hydrophilic.

### 3.5. Surface Roughness

**Figure 6:**
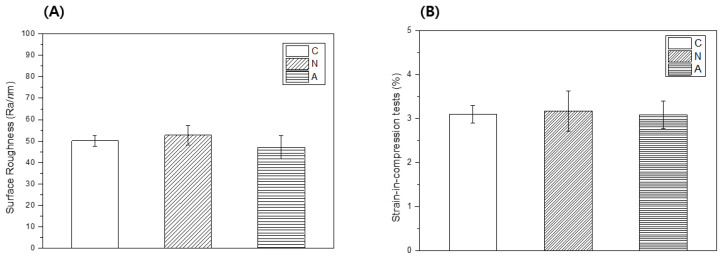
Result of physical properties (**A**) surface roughness of each polyvinyl siloxane impression surface. (**B**) strain-in-compression tests measurement results (%). C: control (nontreated) group, N: nitrogen-plasma-treated group, and A: air-plasma-treated group.

The surface roughness was measured using a 3D optical profiler (Figure 6A). The roughness of the specimen before plasma treatment was 50.09 ± 2.44. The surfaces treated with nitrogen and air NAPPJs showed similar average roughness (R_a_) values of 54.75 ± 15.51 and 46.57 ± 6.33, respectively. There was no significant difference was seen between the plasma-treated and control groups (*p* > 0.05). 

### 3.6. Strain-in-Compression Tests

Figure 6B presents the results of the strain-in-compression tests. The results showed that all tested values were within the range required by ISO 4823 (0.8–20% for light-body material). No significant difference was seen between the control and the plasma-treated experimental groups (*p* > 0.05), with minimum and maximum values of 3.09% and 3.1%, respectively.

### 3.7. Detail Reproduction Test

**Figure 7:**
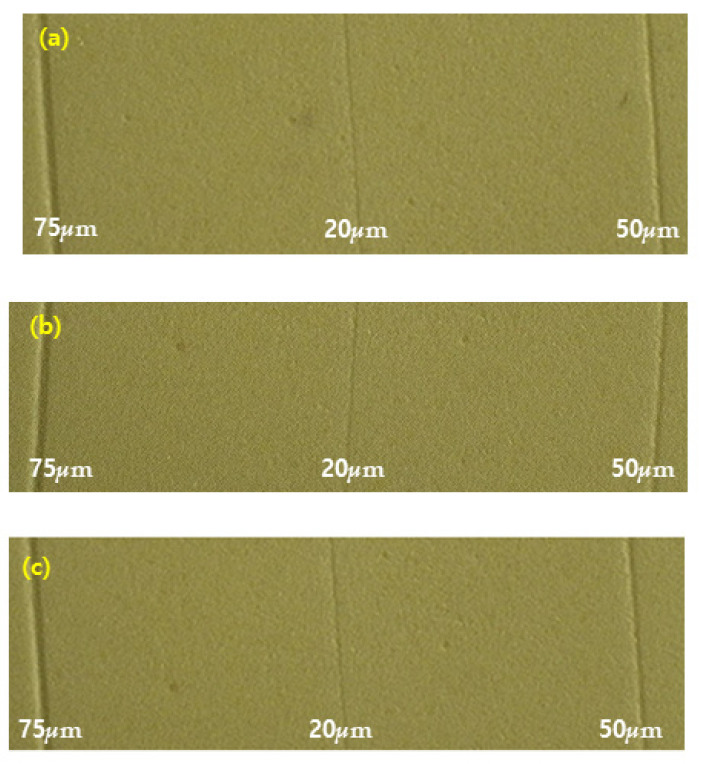
Detailed reproduction test of each polyvinyl siloxane impression surface: (**a**) control (non-treated) group, (**b**) nitrogen-plasma-treated group, and (**c**) air-plasma-treated group.

The results of the detailed reproduction test are shown in Figure 7. No significant difference was seen between the control and test groups of the polyvinyl siloxane impression material. Before and after nitrogen and air NAPPJ treatments with all specimens, the 20, 50, and 75 μm lines were reproduced sufficiently without the use of magnification. No significant differences were noted among the polyvinyl siloxane impression materials with and without nitrogen and air NTAPPJ.

## 4. Discussion

Infection control has gained importance owing to the increased awareness of infectious diseases and the potential for transmission of infectious microorganisms during dental procedures. In particular, dental impressions can easily become contaminated with patients’ saliva and/or blood and can cross-contaminate the stone casts poured against them. Therefore, a wide variety of disinfectants for impressions are commercially available; however, specific recommendations about which one to use are primarily based on the disinfection characteristics of individual disinfectants, because all impression materials are not compatible with all types of disinfectant [17]. Several studies have shown that disinfection procedures affect the physical properties of impression materials (e.g., surface roughness, wettability, dimensional accuracy) [17,18,19].

Recently, plasma disinfection has been introduced as an alternative method. It appears to have advantages over other disinfection methods. Its advantages include the fact this technique is effective in inactivating the required microbial load even at room temperature, and no toxic gases are used. The inactivation of bacteria, biofilm, yeast, and spores is reportedly also possible [20,21]. Therefore, this study aimed to evaluate the antibacterial effect and physical and chemical properties of polyvinyl siloxane impression materials treated with NAPPJ.

First, we evaluated the antibacterial effect. When plasma treatment was applied, the number of bacteria was significantly reduced (Figure 2). This antibacterial effect may have been caused by the ions that are present in the plasma or through some reactive species generated when plasma reacts with O_2_ and N_2_ in the surrounding air [22]. These results are consistent with previous findings. Nitrogen and air NAPPJ treatment was could inhibit *S. mutans* dental biofilm [12,23]. In addition, previous research demonstrated that plasma treatment was more effective in killing both *E.* *coli* and *B. subtilis* compared with UV sterilizer [9].

In this study, the antibacterial effect was also demonstrated through SEM images (Figure 3). The cell membrane of *S. mutans* was damaged as a result of the ionic attack by the plasma. Many studies have investigated plasma disinfection and taken SEM images in which damaged and broken cell membranes can be clearly seen [21,24]. This result suggests at least two types of plasma impacts: first, severe and blanket damage to the physical structure of microorganisms that is likely to cause rapid cell death, and second, less severe punctures that may trigger an irreversible sequence of physical and biochemical changes leading to more gradual cell death [25].

XPS analysis was used to confirm the chemical changes on the plasma-treated surface (Figure 4). Changes in surface chemistry are related to the effectiveness of bacterial adhesion [8,12]. Consistent with other studies, XPS analysis revealed that hydroxyl-related ions, such as OH^−^ and COOH^−^ were increased after both the nitrogen and compressed air NAPPJ treatments. These radicals might improve surface wettability, and they have high oxidation potential owing to the bactericidal effect [26,27]. Furthermore, O_2_ binds with H ions in aqueous solutions to form OH. As this radical is neutral, it can pass through the cell membrane easily and is considered to have antibacterial effects [24,28]. XPS results showed that the peak of C1s decreased following NTAPP treatment. The carbon results from the unavoidable attachment of carbon-containing atmospheric components on the surface, where carbon is normally confirmed to the surfaces [29]. However, the adsorption of carbon on the surface can cause negative biological activity; therefore, it must be reduced for biological activity [29,30]. 

The wettability of impressions is another important physical property because it has been shown to be related to the number of bubbles that form in dies poured from the material [18,31]. After plasma treatment, the contact angles of the experimental groups were lower compared with that of the control (Figure 5). This occurred most likely because the plasma treatments generated free radicals in the materials. Chemical reactions occurring between these free radicals and species, such as atomic hydrogen or oxygen from a polymer surface, incorporate hydrophilic groups to the polymer surface and reduce the contact angle. [26]. The low contact angle produced a dental cast with few voids; therefore, an accurate restoration was achieved [18,32].

All impression materials are not compatible with all types of disinfectants, and the potential for disinfectants to modify the properties of the impression material, such as surface roughness, is high [19]. In this study, the average roughness values of the surfaces with the nitrogen NAPPJ (R_a_ = 54.75 ± 15.51) and air NTAPPJ (R_a_ = 46.57 ± 6.33) before and after NTAPPJ treatment were similar (Figure 6A). It may indicate that additional plasma disinfection chemical reactions did not affect physical properties such as the roughness of impression material.

Impressions should be flexible enough to allow their easy removal from the mouth once set [33]. In ISO 4823, the strain-in-compression of Type 3 impression materials is regulated to between 2% and 20% [34]. In this study, the strain-in-compression of the control group was 3.1%; this satisfied the ISO 4823 standard, and the groups that were treated with plasma also comply with the standard (Figure 6B).

We also measured the detail reproduction test to evaluate the physical change in the impression material (Figure 7). All experimental groups and the control group were able to replicate 20, 50, and 75 *μ*m lines, therefore demonstrating that the plasma treatment does not influence the detail reproduction test. This satisfies the requirement [34]. A lack of surface detail reproduction on the die is one manifestation of a compatibility problem. In this study, the hydrophilic characteristics of the impression materials might make it easy to pour a bubble-free stone cast and enable producing a no-defect stone cast [35,36,37].

This study considered the antibacterial effects of *S. mutans* of NAPPJ on the impression surface. The limitation of this study is that a variety of pathogenic microorganisms including viruses such as hepatitis B or C or herpes simplex viruses, which cause cross-infection in dental clinics, were not evaluated. Furthermore, further in vivo or clinical studies are needed to fully elucidate the efficacy and applicability of NAPPJ treatment. Despite this limitation, we believe that NAPPJ can serve as an effective disinfection tool for dental impression materials.

## 5. Conclusions

When polyvinyl siloxane impressions were subjected to NAPPJ treatment, this study demonstrated the following:

(1) The chemical properties in the impression surface such as surface wettability and chemical composition were changed by NAPPT treatment. However, the physical properties such as surface roughness, strain-in-compression, and detail reproduction were not changed.

(2) The number of bacteria was significantly reduced by NAPPT treatment.

Conclusively, the disinfection effect was observed and the physical properties of the impressions remained unchanged. Therefore, a NAPPJ can serve as an effective disinfection tool for dental impression materials.

## Data Availability

Not applicable.
